# Presenting Patterns of Genetically Determined Developmental Encephalopathies With Epilepsy and Movement Disorders: A Single Tertiary Center Retrospective Cohort Study

**DOI:** 10.3389/fneur.2022.855134

**Published:** 2022-06-20

**Authors:** Mario Mastrangelo, Serena Galosi, Serena Cesario, Alessia Renzi, Lucilla Campea, Vincenzo Leuzzi

**Affiliations:** ^1^Child Neurology and Psychiatry Unit, Department of Human Neuroscience, Sapienza University of Rome, Rome, Italy; ^2^Department of Dynamic and Clinical Psychology, and Health Studies, Sapienza University of Rome, Rome, Italy

**Keywords:** developmental and epileptic encephalopathies, movement disorders, neurogenetic disorders, next generation sequencing-NGS, phenotypes

## Abstract

**Background:**

This paper aimed to evaluate the frequency of observation of genetically determined developmental encephalopathies with epilepsy and movement disorders in a specialistic center, the distribution of etiologies and presenting clinical hallmarks, and the mean times for the achievement of molecular genetic diagnosis.

**Patients and Methods:**

Retrospective data about clinical phenotypes, etiology, and diagnostic pathways were collected in all the genetically confirmed patients with developmental encephalopathies with epilepsy and movement disorders referred to our institution between 2010 and 2020. The cohort was divided into two groups according to the predominant movement disorder type: 1) Group A: patients with hyperkinetic movement disorders; 2) Group B: patients with hypokinetic movement disorders. Both groups were analyzed in terms of developmental, epileptic, and movement disorder phenotypes.

**Results:**

The cohort included 69 patients (Group A = 53; Group B = 16). The etiological spectrum was heterogeneous with a predominance of Rett and Angelman syndrome in Group A and neurodegenerative disorders in Group B. A moderate/severe intellectual disability was assessed in 58/69 patients (mean age at the first signs of developmental impairment = 1,87 ± 1,72 years). Group A included patients with an earlier onset of epileptic seizures (2,63 ± 3,15 vs. 4,45 ± 5,55 years of group B) and a predominant generalized motor semiology of seizures at the onset. Focal seizures were the main initial epileptic manifestations in Group B. Seizures were noticed earlier than movement disorders in Group A while the opposite occurred in Group B. A higher increase in molecular genetic diagnosis was obtained in the last five years. Mean diagnostic delay was longer in Group B than in Group A (12,26 ± 13,32 vs. 5.66 ± 6.41 years). Chorea as an initial movement disorder was associated with a significantly longer diagnostic delay and a higher age at etiological diagnosis.

**Conclusions:**

This study suggested: (a) a higher frequency of genetic defects involving neurotransmission, neuronal excitability, or neural development in patients with hyperkinetic movement disorders; (b) a higher frequency of neurodegenerative courses and a longer diagnostic delay in patients with hypokinetic movement disorders.

## Introduction

The spectrum of genetic developmental encephalopathies presenting with epilepsy and movement disorders has significantly expanded in the last decade with the characterization of more than 100 monogenic disorders, and several genotype-phenotype correlation studies prominently focusing on single genes, specific pathogenic copy number variants, or non-mendelian conditions (1, 2).

The increasing availability of next generation sequencing techniques resulted in a higher number of etiological diagnoses, but also highlighted the importance of a careful evaluation of phenotypes for the interpretation of molecular data (3, 4).

The present single tertiary center study retrospectively analyzed the distribution of etiologies and presented phenotypes in a cohort of patients with these complex pediatric-onset disorders. The study aimed to evaluate the frequency of observation of these conditions in a specialistic center, their most frequent clinical hallmarks, and the mean times for the achievement of molecular genetic diagnosis in the last decade (during the age of introduction and diffusion of next generation sequencing methods).

## Patients and Methods

We retrospectively collected 69 patients (35 females and 34 males) with genetically confirmed developmental encephalopathies with epilepsy and movement disorders, who were referred to our Institution and received a genetic diagnosis between January 2010 and January 2020.

Medical records and video recordings for movement disorder characterization were reviewed and data were collected in a standardized digital form to include demographic information, perinatal history, developmental milestones, predominant seizure, and movement disorder subtypes, onset and evolution of seizures and movement disorders during the follow-up, the severity of developmental impairment, age at the diagnosis, diagnostic delay (time between the onset of symptoms and the molecular genetic diagnosis), EEG patterns at onset and during the follow-up, neuroimaging features, relevant neurophysiologic and laboratory investigations, pathogenic or likely pathogenic gene or copy number variants.

Seizure types and epilepsy syndromes were classified according to the 2017 classification by ILAE Commission for Classification and Terminology (5, 6).

Patients were subdivided into two groups, patients with hyperkinetic (Group A) and patients with hypokinetic (Group B) movement disorders according to the same criteria that are currently adopted for adults (7). Hyperkinetic movements (i.e., unwanted and excess movements) included dystonia, chorea, athetosis, myoclonus, tremor, tics, ataxia, and stereotypies. Hypokinetic movements (i.e., decrease in the number of movements) included hypokinetic-rigid syndrome or parkinsonism (8, 9).

In both groups, we evaluated the timing for the achievement of the etiological diagnosis through a comparison between temporal parameters (age at the onset of seizures, age at the onset of movement disorders, age at the molecular genetic diagnosis and diagnostic delay) and clinical manifestations (seizure types and type of movement disorder).

We also analyzed the distribution of the diagnostic delay during the years in which the molecular diagnoses were made. The temporal periods were divided in 4 intervals: PERIOD 1: 2016-2020; PERIOD 2: 2011- 2015; PERIOD 3 = 2006-2010; PERIOD 4 = before 2005. This last temporal clustering was introduced to minimize the burden of the different diagnostic yields associated with the various molecular genetic technologies that were available over the years. These different methods included Sanger sequencing of specific single genes, array CGH, targeted gene panels for epilepsy and/or movement disorders, clinical exome, or whole-exome sequencing. Single gene variants were classified according to the American College of Medical Genetics criteria (3, 10).

Statistical analysis was performed using SPSS 26.0 statistical software package for Windows. Data were reported as frequencies and percentages for discrete variables, as well as means and standard deviations for continuous variables. Chi-squared test (χ2) was used to reveal differences between and within groups in the discrete variables investigated, while a one-way analysis of variance (ANOVA) was carried out to test between and within groups differences in continuous variables. Bonferroni correction was applied to the *post-hoc* tests. The significance level for all statistical tests was set a priori to α = 0.05.

## Results Cohort Composition

Sixty-nine patients (35 females and 34 males), with a mean age of 16.26 ± 9.18 years, with a confirmed molecular genetic diagnosis, were selected. Group A included 53 patients (29 females and 24 males) while 16 patients (6 females and 10 males) belonged to Group B.

The mean age at the molecular genetics diagnosis was 8.75 ± 8.743 years with a mean diagnostic delay of 6.21 ± 8.05 years.

Tables 1, 2 summarize the demographic, genetic, and clinical data of patients with hyperkinetic (Group A) and hypokinetic movement disorders (Group B).

**Table 1:** Demographic, clinical, and molecular genetics features of patients with developmental encephalopathies presenting with epilepsy and hyperkinetic movement disorders (Group A).

**Predominant movement disorder**	**Demographic data**		**Etiology**			**Age at onset of symptoms and time to diagnosis**					**Symptoms at the onset**			**Symptoms during the follow-up**		
	**Patient and sex**	**Age**	**Diagnosis**	**Gene**	**Gene variant**	**Age at the diagnosis**	**Age at the onset of seizures**	**Age at the onset of movement disorders**	**Age at onset of neuro-developmental disorder**	**Diagnostic delay**	**Seizure type at the onset**	**Movement disorder at the onset**	**Neuro-developmental disorder signs at onset**	**Seizure** **type during the follow-up**	**Movement disorder during the follow-up**	**Neuro-developmental disorder during the follow-up**
**Chorea**	1 M	34	GNB1 encephalopathy	*GNB1*	c.357C> G (p.Asn119Lys)	34	1	8	NA	33	FIA,FA,M,A,TC	Ch, D (p)	NA	SF	Ch,D,P	SP-ID
	2 M	26	KCNA2 encephalopathy	*KCNA2*	c.890G > A (de novo)	23	2	1	24	21	FS, TC	Ch, Ata	Clu	SF	Ata, D	Mo-ID
	3 M	16,58	MeCP2 duplication syndrome	MeCP2	dup Xq28	2,1	1,08	11	10	1,18	TC	Ch, S	HCD	TC, A, T, C	S	SP-ID
	4 M	15,16	MeCp2 duplication syndrome	MeCP2	dup Xq28	4	9	12	24	5	T, A	Ch	W	T	S	SP-ID
	5 M	9,7	MEF2C encephalopathy	*MEF2C*	microdel 5q14.3	10,83	1,4	1,5	17	9,4	FTBTC	Ch	TCD	FA, T	Ch	SP-ID
	6 M	9,3	GNAO1 encephalopathy	GNAO	c139 A > G	2,75	4	5	9	2,5	FIA	Ch, D, B (p)	HCD	FIA, FTBC	D, S, B	SP-ID
**Ataxia**	7 M	27,6	Succinic semialdehyde dehydrogenase deficiency	ALDH5A1	0.526G > A /c.278>T	5	0,58	11	8	4,4	A	Ata	HCD	A, C, TC	Ata	Mo-ID
	8 F	21,25	BRAT 1-related syndrome	BRAT1	c.638_639insA/ c.1395G>A	19	13,9	13,9	36	5,08	T	Ata	LDD	FTBC	Ata	Borderline
	9 F	20,3	KCTD7-related progressive myoclonus epilepsy	KCTD7	c.533C>T	6,4	0,8	1,6	18	5,5	M	Ata	R	TC, A, AA	Ata	SP-ID
	10 F	18,1	Ceroid lipofuscinosis type V	CLN5	c.595 C > T	9,6	5	5	NA	4,6	C	Ata	R	C	D	SP-ID
	11 F	18	Angelman syndrome	15q11-q13	PWS-AS maternal allele absence	2	1	10,83	24	1	FIA	Ata	LD	A, FIA	Ata	SP-ID
	12 F	14,4	Angelman syndrome	15q11.2-12	altered methylation fragments SNRPN 04103, 04106,04104,11181	10,8	10	1,5	12	9,3	AA	Ata	LWD	My	Ata	SP-ID
	13 F	14,3	GLUT1-deficiency	SLC2A1	c.470dup	11	3	0,1	24	8	AA	Ata (p)	LWD	AA	Ata, D	M-ID
	14 M	13,5	GLUT 1 deficiency	SLC2A1	c.631 C > T	9	2	5	36	7	AA	Ata (p)	AD	M, TC	Ata	Mo-ID
	15 F	13	SCN1A encephalopathy	*SCN1A*	c.4907G > A; p.Arg1636Gln	8	0,2	7	36	8	C, T, TC	Ata	W	SF	Tr,P,Ata	Mo-ID
	16 F	12,3	GLUT1 deficiency	SLC2A1	c.274 > T	3	0,33	0,66	NA	2,6	FIA	Ata (p)	LDD	FIA	Ata	Mo-ID
	17 M	12,1	Angelman syndrome	15q11q13	15q11q13del	12,4	2	2	24	10,4	AA	Ata	LDD	AC, C	Ata	SP-ID
	18 M	11,3	Ceroid lipofuscinosis type II	TPP1	c.225A > G/c.1542A > T	4,25	3	3	18	1,25	FTBTC	Ata	LWD	FTBC	Ch	SP-ID
	19 F	5,3	Ceroid lipofuscinosis type II	TPP1	c.1644G > A	4	3	4	40	1	A	Ata	LR	A	Ata	ASD
**Dystonia**	20 M	32,16	Succinic semialdehyde dehydrogenase deficiency	ALDH5A1	c.526G > A /c.278 > T	11	7	3	36	4	C	D, S	LDD	AA, TC	D, S	ASD
	21 F	17,25	Niemann Pik type C	NPC1	c.349 G > A/c2795+a G > C	4	4	2,3	60	1,6	TC	D. S	AD, R	M, T, FA	D, Br	SP-ID
	22 F	14,9	Rett syndrome	CDKL5	Xp22.13 DEL	6	0,08	1,4	11	5,9	FA	D	R	TC, FTBC, T, C	D	SP-ID
	23 M	14,6	MFF encephalopathy	MFF	c.892 C > T	11	1,5	1,5	12	9,5	M	D	TCD	FA	D	SP-ID
	24 M	14	SCN8A paroxysmal dyskinesia with epilepsy	*SCN8A*	c.4447G > A; p.E1483K	14	9 months	13	84	12	FIA,FA	D (p)	Clu	SF	D, P	Borderline, DCD
	25 M	13,9	Niemann Pick type C	NPC1	c.1211G > A/c.3493G > A	10	10	10,25	84	0	FIA	D, S	AD, R	AA, C, A, FTBC	D	SP-ID
	26 F	13,8	PRRT2-related syndrome	PRRT2	c.649dup	10	0,4	1,1	18	9,5	T	D	LWD	AA, T, M	Ata	SP-ID
	27 M	13,6	KDM5C- encephalopathy	KDM5C	c.1592C > T	8,6	10	7	4	2	TC	D	LD	TC	D	M-ID
	28 M	12,6	ATP1A3 encephalopathy	ATP1A3	c.2324C > G	7	0,25	0,25	24	6,75	T, AA	D (p)	LWD	AA, T	D, Ata	SP-ID
	29 F	10,5	CHD2 encephalopathy	CHD2	c.561del	9	0,66	4	24	8,3	FIA	D	LDD	FTBC, A, C	D	M-ID
	30 F	9,7	PMM2 encephalopathy	PMM2	c.323C>T/ c.710C>G	9	0,4	0,4	20	8,5	FIA	D	TCD	FA	Ata,	SP-ID
	31 M	9,7	Canavan disease	ASPA	G503	1,8	0	1,8	6	1,8	T	D	HCD	C	D, R, My	SP-ID
	32 F	8,2	AP4M1-related syndrome	AP4M1	c.10 C > T/ 498del	6	0,58	0,58	6	5,4	A	D	HCD	AA	D	SP-ID
	33 F	4,6	FOXG1 encephalopathy	FOXG1	c.946del	1,3	0,58	0	24	0,75	FIA	D	TCD	FTBC	D	GDD
	34 M	4	Sodium cluster channel deletion developmental encephalopathy with epilepsy	2q24.3q31.1	Microdeletion on 2q24.3q31.1, (164375953-	1,5	0,5	0,33	8	1	TC	D	TCD	AA, C	D, Ch	GDD
	35 F	2,5	PRRT2-related syndrome	PRRT2	microdel 16p11.2	0,6	0,4	0,66	24	0,25	FTBTC	D	LDD	FTBC, A	D	GDD
**Myoclonus**	36 M	28	ARGHEF9	*ARGHEF9*	c.1300G > C (de novo)	24	1,6	3	18	22,4	FS,TC	Hy, My	LDD	SF	Hy,Tr,D	Mo-ID
**Stereotypes**	37 F	29,5	Rett syndrome	MeCP2	803delG	7	7	2	20	5	A	S	R	TC	S	SP-ID
	38 F	19,5	FOXG1-encephalopathy	FOXG1	c.969delC	7	0,58	0,33	4	6,4	T	S, D	LWD	T	Ch	SP-ID
	39 F	18,08	Rett syndrome	CDKL5	c.587C > T	5	0,08	1	15	4,9	C	S	TCD	T, TC	S	SP-ID
	40 M	16,3	SCN1B-related epilepsy	SCN1B	c.574G > A	11,1	0,8	0,8	12	10,3	FTBTC	S	LDD	FTBC, TC	S	ASD
	41 F	14,6	Angelman syndrome	15q11-q13	paternal uniparental disomy (15q11-q13), altered methylation of maternal allele	3	3	5	21	0,12	M	S	LWD	AA	S	SP-ID
	42 F	14	PURA encephalopathy	PURA	c.768 dup	11	0,9	0,8	3	10,1	T	S	LH	AA, T, A	s	SP-ID
	43 F	13	Rett syndrome	CDKL5	c.551 T > A	2,3	0	2,08	NA	2,33	T	S, A	W	T, IS, TC	D, Ata, S	SP-ID
	44 F	12,25	Rett syndrome	MeCP2	c.502C > T	1,6	1,5	1,75	22	0,16	FIA	S	TCD	FA	S	SP-ID
	45 F	12	Angelman syndrome	15q11.2-12	15q11.2-12 del	3,8	3,8	4	5	0	TC	S	HCD	C	S, My	SP-ID
	46 M	11	Angelman syndrome	15q11-q13	del15q11-q13	1,5	0,91	1,25	12	0,58	C	S	TCD	C, A, TC	S, Tr	SP-ID
	47 F	10,6	Rett syndrome	MeCP2	c.808C > T	4,5	1,5	1	18	0,25	T	S	W	SF	S	SP-ID
	48 F	10,5	IQSEC2-encephalopathy	IQSEC2	c.4110_4111del	6	3	2	24	2,75	T	S	LDD	FA	S	ASD
	49 M	10,4	IQSEC2 encephalopathy	IQSEC2	c.854del	6	2	3	15	4	FTBTC, A	S	TCD	AA, FIA	S, D	SP-ID
	50 F	7	Rett syndrome	MeCP2	c.445.C > G	4	6,3	1,6	18	2,3	T	S	LH	T	S, Ata	SP-ID
	51 F	7	SYNGAP1 encephalopathy	*SYNGAP1*	c.3706C > T	3,6	4	1	15	none	AA, FIA, M	S	TCD	AA, A	S,D,Ata	ASD
	52 M	5,08	PRICKLE1 encephalopathy	PRICKLE1	c.820G > A	1,1	0,83	1	12	0,33	C	S	W	T, FA	S	SP-ID
	53 M	3,1	Williams syndrome	7q11.23	microdeletion 7q11.23	1,6	1,4	1,25	10	0,16	A	S	HCD	T	S, My	SP-ID

*T, tonic; A, atonic; AA, Atypical absences; Br, bradykinesia; FTBTC, FA to bilateral tonic clonic; M, myoclonic; FIA, focal seizures with impaired awareness; FA, focal seizures with preserved awareness; C, clonic; D, Dystonia; Ata, ataxia; S, stereotypies; B, ballismus; Ch, chorea; My, myoclonus; p, paroxysmal; R, rigidity; IS, infantile spasms; Tr, Tremor; TC, tonic-clonic; Hy, hyperekplexia; FS, febrile seizures; SF, seizure free; HCD, head control delay; TCD, trunk control delay; LDD, language development delay; LD, language disorder; LR, language regression; AD, academic difficulties; R, psychomotor regression; Clu, clumsiness; LWD, language and walking delay; W, walking delay; LH, language halt; DCD, developmental coordination disorder; Borderline, Borderline cognitive impairment; M-ID, Mild intellectual disability; Mo-ID, moderate intellectual disability; SP- ID, severe/profound intellectual disability; ASD, Autism Spectrum Disorder; GDD, global developmental delay; NA, not available*.

**Table 2:** Demographic, clinical, and molecular genetics features of patients with developmental encephalopathies presenting with epilepsy and hypokinetic movement disorders (Group B).

**Predominant movement disorder**	**Demographic data**		**Etiology**			**Age at diagnosis and age at onset of symptoms**					**Symptoms at the onset**			**Symptoms during the follow-up**		
	**Patients - sex**	**Age (years)**	**Diagnosis**	**Gene or cromosomal region** **Gene variant o cnv**		**Age at diagnosis (years)**	**Age at the onset of seizures**	**Age at the onset of movement disorder**	**Age at the onset of neuro-developmental disorder**	**Diagnostic delay**	**Seizure type at the onset**	**Movement disorder at the onset**	**Neuro-developmental disorder signs at onset**	**Seizure type during the follow-up**	**Movement disorder during the follow-up**	**Neuro-developmental disorder during the follow - up**
**Hypokinesia**	1 F	36,91	WDR45 deficiency	*WDR45*	c.439+5G > A	35	5	5	NA	30	TC	H, P	NA	SF	P, D	SP-ID
	2 M	29,73	HIBCH deficiency	*HIBCH*	C.777T > A	25	6	0	6	19	M	H, Ata, My	H	SF	D, H, Tr	SP-ID
	3 F	17,9	DHPR deficiency	*QPDR*	c.41T > C	11,8	3	2	36	8,3	AA	H, Ata, D	LD	FIA, C, TC	P, D	SP-ID
	4 M	13	Menkes disease	*ATP7A*	c.3561G > A	0,5	0,41	0,41	NA	0,08	FIA	H	NA	AA	H	SP-ID
	5 M	8,4	Menkes syndrome	*ATP7A*	c.2938C > T	1	1	0,66	6	0	FIA	H	R	FIA	P	SP-ID
**Parkinsonism**	6 F	43	Rett syndrome	*MECP2*	c.547A > T	2,08	1,5	1	NA	43	T	S	NA	AA	S, H	SP-ID
	7 M	36,9	KCND3 encephalopathy	*KCND3*	c.901T > C	17	7	5	36	10	FIA	T, D	Clu	FIA	P, D	SP-ID
	8 M	35,15	DHDDS deficiency	*DHDDS*	c.632G > A	35	10	3	18	25	FIA	T, My	TCD	M	P, My	ASD
	9 F	30	KCNQ2 encephalopathy	*KCNQ2*	c.629G > A	30	0	2	NA	30	FIA	S, H	NA	FIA, TC	D, S, H	SP-ID
	10 M	22,9	Neuronal ceroid lipofuscinosis type 6	*CLN6*	c.700 T > C	13,5	13	13	NA	0,5	FIA	My, P	NA	TC	P	SP-ID
	11 F	22	Rett syndrome	*MeCP2*	c.473 C > T	8	4	2	18	44	FIA	S, P	LR	TC	S, H	SP-ID
	12 M	21	Dravet Syndrome	*SCN1A*	C.4814A > T	5	0.5	2.6	120	4.6	C	Tr	AD	T, C, TC	P,Tr	M-ID
	13 F	17	SYNGAP1 encephalopathy	*SYNGAP1*	c.1352 T > A	16	14	15	36	3	AA, M	Tr	LDD	AA, M	P, D, T, Ata	Mo-ID
	14 M	13	WARS2- early onset parkinsonism	*WARS2*	WARS2 c.37T > G and c.679A > G	12,5	5	0,91	13	11,5	FIA	P, T, My, D	R	FIA	P, My, D, Tr	SP-ID
	15 M	9,9	Menkes disease	*ATP7A*	del c.467 delA	0,6	0,66	0	6	0	IS	P	H	SF	P	SP-ID
	16 M	8,75	Adenyl-succinate lyase deficiency	*ADSL*	c.65C > T and c.340 T > C	6,4	0,25	0	6	6,16	FIA	P	H	T, M, TC	P	SP-ID

*FIA, focal seziures with impaired awareness; S, Stereotypes; TC, Tonic-clonic; H, hypokinesia; M, Myoclonic; AA, Atypical absences; IS, Infantile spasms; Ata, Ataxia; C, clonic; SF, seizure free; D, dystonia; P, Parkinsonism; Tr, Tremor; My, myoclonus; NA, not available; HCD, head control delay; TCD, trunk control delay; LDD, language development delay; LD, language disorder; LR, language regression; AD, academic difficulties; R, psychomotor regression; Clu, clumsiness; LWD, language and walking delay; W, walking delay; LH, language halt; DCD, developmental coordination disorder; Border, Borderline cognitive impairment; M-ID, Mild intellectual disability; Mo-ID, moderate intellectual disability; SP- ID, severe/profound intellectual disability; ASD, Autism Spectrum Disorder; GDD, global developmental delay*.

Table 3 summarizes both groups: (a) demographic data; (b) temporal distribution of clinical presentations (e.g., age at the onset of each cluster of symptoms) and diagnostic steps (e.g., age at the diagnosis and diagnostic delay); (c) distribution of seizure and movement disorder types.

**Table 3:** Differences between groups in continuous and discrete variables.

	**Group A** ***N*** **=** **53 (29 F; 24 M)**		**Group B** ***N*** **=** **16 (6F; 10M)**		**F**	***p*-value**
	**M**	**SD**	**M**	**SD**		
Age (years)	14,04	7,11	23,62	11,92	15.849	0.001
Age at the diagnosis (years)	7,48	6,34	16,32	13,61	13.203	0.001
Age at the onset of seizures (years)	2,63	3,15	4,45	4,55	3.280	0.075
Age at the onset of movement disorders (years)	3,50	3,75	3,55	4,55	0.01	0.974
Age at the diagnosis of neurodevelopmental disorder (years)	2,28	2,76	1,78	1,41	0.744	0.392
Diagnostic delay (years)	5,66	6,41	12,26	13,32	7,467	0.008
	***N***	**%**	***n***	**%**		
Gender					1.458	0.265
M	24	45.25	10	62.5		
F	29	54.75	6	37.5		
Seizure types					15,353	0.004
Generalized motor	27	50,9	5	31,25		
Generalized non motor	5	9.4	-	-		
Focal to bilateral tonic clonic	5	9.4	-	-		
Focal motor	10	18,8	11	68,75		
Focal non-motor	6	11,3	-	-		
Movement disorder type					44.167	0.001
Ataxia	13	24,5	1	6,25		
Dystonia	17	32,07	1	6,25		
Parkinsonism	-	-	5	31.25		
Stereotypies	17	32,07	3	18,75		
Hypokinesia	-	-	3	18,75		
Tremors	-	-	3	18,75		
Chorea	4	7.5	-	-		
**Neurodevelopment disorder type**						
Developmental coordination disorder	1	1	-	-		
Borderline cognitive impairment	3	6	-			
Global developmental delay	3	6	-	-		
Mild intellectual disability	3	6	1	3		
Moderate intellectual disability	5	9	1	3		
Severe and profound intellectual disability	33	62	13	91		
Autism spectrum disorder	5	9	1	3		

*Significance = p < 0.01*.

In Group B the mean patient age was almost double compared to that of Group A (Table 3).

A neurodegenerative course was observed in 30/53 patients of Group A and 14/16 patients of Group B.

## Etiological Spectrum

**Figure 1:**
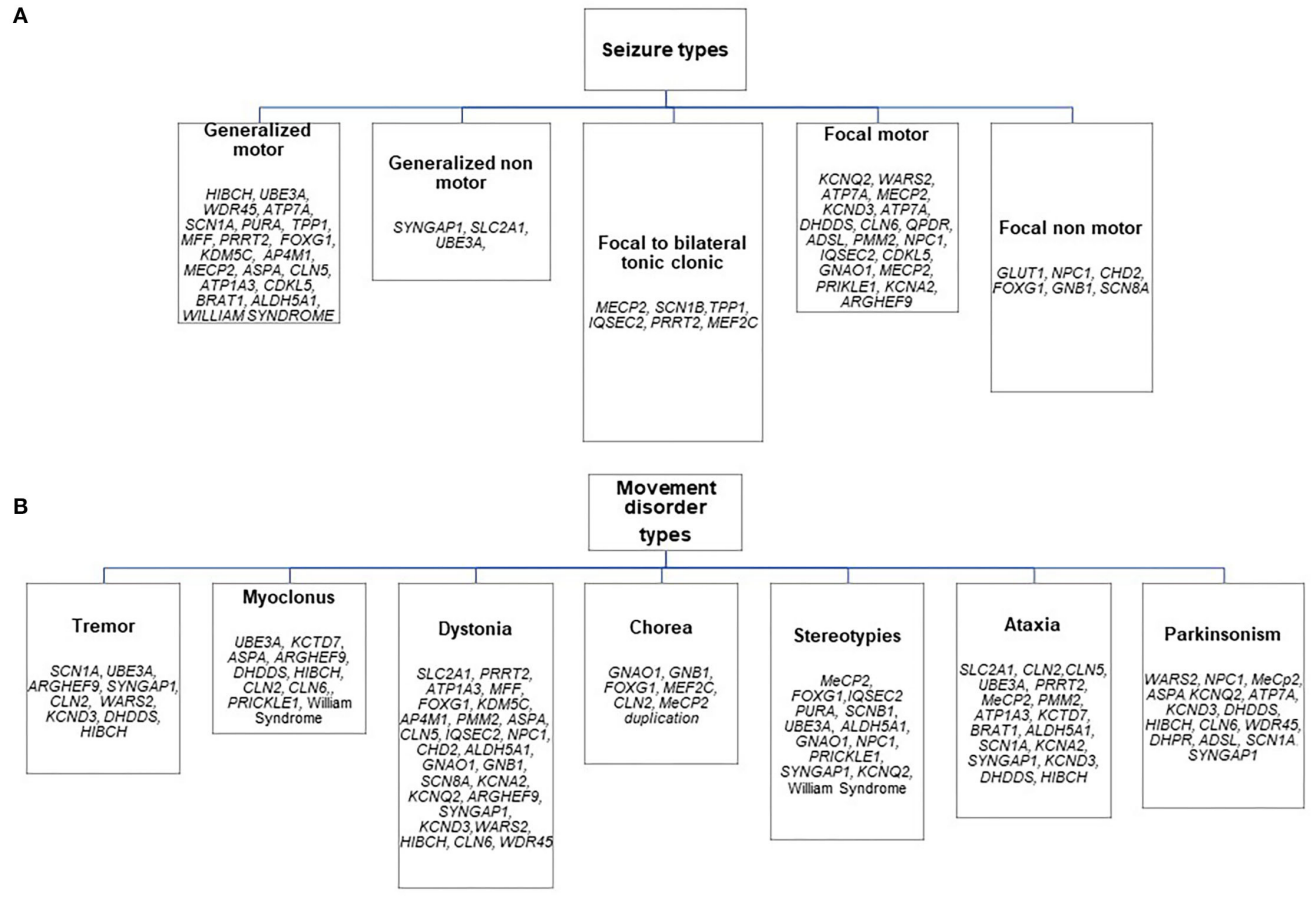
Distribution of genetic etiologies according to presenting seizure **(A)** and movement disorder **(B)** types in the whole sample.

The distribution of genetic etiologies according to the different presenting seizure and movement disorder types in the whole cohort is summarized in Figure 1, Table 4.

**Table 4:** Distribution of epilepsy and movement disorder phenotypes at onset and during the follow-up in the different functional groups of genetic etiologies.

**Etiological category**	**Genes**	**N of patients**	**Movement disorder phenotype at onset (n of patient)**	**Movement disorder phenotype on follow up**	**Seizures phenotype at onset (n of patient)**	**Seizures phenotype on follow up**
Channellopathies	*KCTD7, KCNQ2, KCNA2, KCND3, SCN1A, SCN1B, SCN8A, 2q24.3q31.1 deletion*	9	Dystonia (3/9), Ataxia (2/9), Stereotypies (2/9), Chorea(1/9), Tremors (1/9)	Dystonia 4/9, Ataxia (2/9), Parkinsonism (1/9), Tremors (1/9), Stereotypies (1/9)	Generalized motor (4/9), Focal motor (3/9), Focal to bilateral tonic-clonic (1/9), Focal non motor (1/9)	Generalized motor (5/9), Focal motor (2/9), Focal to bilateral tonic-clonic (1/9), Focal non motor (1/9)
Transportopathies	*SLC2A1, ATP1A3, ATP7A*	7	Ataxia (3/7), Hypokinesias (2/7), Dystonia (1/7), Parkinsonism (1/7)	Ataxia (2/7), Dystonia (2/7), Parkinsonism (1/7), Hypokinesias (1/7) Stereotypies (1/7)	Generalized motor (2/7), Generalized non motor (2/7), Focal Motor (2/7), Focal non motor (1/7)	Generalized non motor (4/7), Focal motor (1/7), Focal non motor (1/7), seizure free (1/7)
Synapthopathies	*PRRT2*	2	Dystonia (2/2)	Chorea (1), Dystonia (1)	Generalized motor (1/2), Focal to bilateral tonic-clonic (1/2)	Generalized non motor (1/2), Focal to bilateral tonic-clonic (1/2)
Disorders of intermediate metabolism	*DHPR, ALDH5A1, HIBCH, ASPA, ADSL, WARS2*	7	Dystonia (2/7), Ataxia (2/7), Parkinsonism (2/7), Hypokinesias (1/7)	Parkinsonism (3/7), Dystonia (3/7), Ataxia (1/7)	Generalized motor (4/7), Focal motor (3/7)	Generalized motor (3/7), Generalized non motor (1/7), Focal to bilateral tonic-clonic (1/7), Focal motor (1/7), seizure free (1/7)
Disorders of complex molecule and organelle metabolism	*CLN2, CLN5, PMM2, MFF, WDR45, AP4M1, NPC1, DHDDS*	11	Dystonia (5/11), Ataxia (3/11), Parkinsonism (3/11), Tremors (1/11)	Dystonia (5/11), Parkinsonism (3/11), Stereotypies (2/11), Ataxia (1/11)	Generalized motor (5/11), Focal motor (4/11), Focal non motor (1/9), Focal to bilateral tonic-clonic (1/9)	Generalized motor (5/11), Generalized non motor (2/11), Focal motor (2/11), Focal non motor (1/9), Focal to bilateral tonic-clonic (1/9)
Disorders of post-synaptic cellular signaling	*IQSEC2, GNAO1, GNB1, ARGHEF9, SYNGAP1*	7	Stereotypies (4/7), Tremors (1/7), Chorea (1/7), Myoclonus (1/7)	Stereotypies (3/7), Parkinsonism (1/7), Dystonia (1/7) Chorea (1/7), Hyperklepsia (1/7)	Generalized motor (3/7), Generalized non motor (2/7), Focal non motor (1/7), Focal to bilateral tonic-clonic (1/7)	Generalized motor (2/7), Generalized non motor (3/7), Focal non motor (2/7)
Disorders of cellular cycle's regulation	*FOXG1, PURA, CDKL5, MECP2, CH2D, BRAT1, KDM5C, MEF2C, PRIKLE1*	19	Stereotypies (10/19), Dystonia (4/19), Ataxia (1/19), Chorea (3/19)	Stereotypies (10/19), Dystonia (4/19), Ataxia (2/19), Chorea (2/19)	Generalized motor (10/19), Focal motor (5/19), Focal to bilateral tonic-clonic (2/19), Focal non motor (2/19)	Generalized motor (9/19), Generalized non motor (3/19), Focal motor (2/19), Focal to bilateral tonic-clonic (1/19), Focal non motor (3/19)
Disorders of degradation/turnover of intra and extracellular components	*UBE3A*	6	Ataxia (3/6), Stereotypies (3/6)	Ataxia (4/6), dystonia (1/6), Stereotypies (1/6)	Generalized motor (3/6), Generalized non motor (2/6), Focal motor (1/6)	Generalized motor (4/6), Generalized non motor (1/6), Focal non motor (1/6)

Almost half of the cases carried pathogenic variants of genes involved in the regulation of the cellular cycle and intracellular metabolism (Table 4).

In Group A the most frequent diseases were Angelman Syndrome (6/53) and typical (5/53 patients harboring pathogenic MeCP2 variants) or Atypical Rett Syndrome (8/53 patients with pathogenic variants in *CDKL5, FOXG1, IQSEC2, PURA* or *MEF2C* genes), accounting for 35% of diagnosis (19/53). A metabolic disorder was diagnosed in 22% of patients (13/53). Sodium and potassium channelopathies (5/23 patients), synaptopathies (3/53), and postsynaptic signaling disorders (3/53) accounted for most of the remaining cases.

In Group B 10/16 patients had a metabolic disorder (3/9 with Menkes disease), 3/16 a sodium or potassium channelopathy (*SCN1A, KCNQ2, KCND3*), 2/16 Rett syndrome, and 1/16 a synaptopathy (Table 2). All metabolic disorders described in this group, except for DHPR deficiency, had a neurodegenerative course and 5/16 were associated to brand new genes or genes described in the last decade (e.g., *WARS2, KCND3, DHDDS, HIBCH, WDR45*).

A subset of genes including MeCP2, *SCN1A*, and *SYNGAP1* was identified both in Group A and B in patients with different ages and disease stages (Tables 1, 2). Coherently hypokinetic features have been reported as late features in these conditions, preceded by hyperkinetic movement disorders.

## Developmental History

A complete developmental history was available for 62/69 patients. The mean age at the first signs of neurodevelopmental impairment was lower than the onset of seizures and movement disorders without significant differences between Group A and Group B (Table 3).

No developmental milestones were achieved in 16/69 patients while 48/69 patients experienced different degrees of delay in one or more developmental milestones (Table 3). Motor impairment was variable with an autonomous walking that was achieved in 34/69 patients while 18/69 were wheelchair bounded and 17/69 were bedridden.

A profound-severe to moderate cognitive impairment was diagnosed in 58/69 patients (71% of patients in Group A vs. 94% of patients in Group B). Developmental regression was observed in 8 patients (Patients 9, 10, 19, 21, 22, 25, 37 in Table 1 and patient 14 in Table 2). An autism spectrum disorder was diagnosed in 12% of patients.

The developmental impairment was borderline in 2 patients belonging to group A (patients 8 and 24 in Table 1). Patient 8 in Table 1 was a female carrying a pathogenic variant of *BRAT1* gene who had also a less severe epilepsy phenotype compared to other cases previously reported in the literature harboring the same variant (11). Patient 24 in Table 1 presented with a previously reported *SCN8A* variant associated with a benign childhood focal epilepsy, paroxysmal dyskinesia, and borderline cognitive functioning with minor coordination issues (12).

## Epilepsy Phenotype

Seizure onset occurred during infancy or early childhood in 82,7% of patients. Motor seizures accounted for 82,61% of initial epileptic manifestations with almost half of the patients presenting with a generalized semiology while the frequency of non-motor seizures tended to double on follow-up (Table 4).

Patients belonging to Group A experienced an earlier onset of seizures than the ones of group B even if the difference in terms of age at the onset was not statistically significant (Table 3).

The initial epileptic manifestations mainly included generalized motor seizures in Group A (with a predominance of tonic seizures accounting for 21% of cases) and focal motor seizures with impaired awareness in Group B (56% of the cases) (Tables 1–3). In Group A, seven patients became seizure-free (13,2%), and none in Group B (Tables 1, 2).

These clinical patterns were associated with a concurrent predominance of multifocal (58,5% of patients in Group A) and focal (43% of patients in Group B) interictal EEG abnormalities. EEG was generally non-specific in terms of consistency with the epilepsy phenotype reported in the literature for the considered gene, with two significant exceptions in Group A (Table 1: Patients 9 and 19 who had early onset photosensitivity at low frequencies in association, respectively, with a neuronal ceroid lipofuscinosis type 2 and KCTD7-related progressive myoclonus epilepsy) and in two patients of Group B (Table 2: Patient 9 who had focal abnormalities during neonatal age as frequently reported in KCNQ2 encephalopathy and Patient 15 with Menkes disease who experienced a West syndrome at the age of 8 months).

The response to antiepileptic treatments was poor in most patients with the best response to valproate (27.65% of patients in Group A and 28,57% in Group B). A ketogenic diet resulted in a dramatic seizure reduction in 3 patients with Glut1 deficiency (Table 1: patients 13, 14, and 16) and one patient with adenyl-succinate lyase deficiency (Table 2: patient 16). The treatment with carbamazepine resulted in a mild improvement of paroxysmal dystonia in one patient with ATP1A3 encephalopathy (Table 1: patient 28) while a ketogenic diet was associated with an improvement of ataxic gait in 3 patients with Glut1 deficiency (Table 1: patients 13, 14, and 16). Valproate induced a worsening of tremors in 5 patients (Table 1: patients 13, 36, and 46; Table 2: patients 2 and 13).

## Movement Disorder Phenotype

The onset of movement disorders was noticed in the age-range 1–6 years in 57,97% of cases and before the age of 12 months in 24,63% of patients. A relevant number of patients presented with more than one subtype of hyperkinetic movement disorder (11 out of 53 in Group A) or hyper and hypokinetic features observed simultaneously at disease onset or sequentially during the follow-up (7 out of 16 in Group B). In 7 out of 16 patients belonging to Group B, parkinsonism was preceded by hyperkinetic movement disorders (Patients 2, 4, 6, 7, 9, 11, 13 in Table 2).

Stereotypes and dystonia were the most frequent hyperkinetic movement disorders at the onset in Group A (17 patients for both symptoms: Tables 1, 3). The first ones were predominant in the disorders of the cellular cycle's regulation while dystonia was the presenting signs of different groups of disorders involving all the steps of cellular signaling and metabolism (Figure 1; Table 4). Paroxysmal movement disorders were observed at the onset in 3 patients with GLUT1 deficiency who presented with episodic ataxias and in 1 patient with ATP1A3-related dystonic attacks (Patients 13, 14, 16, and 28 in Table 1).

In Group A, only 2 patients (Patients 6 and 18 in Table 1) received pharmacological treatments for movement disorders with a limited response, while a few therapeutic strategies were attempted in Group B in which the most relevant positive effects were observed in 2 patients (Patient 3 and 14 in Table 2) who received dopaminergic agents to treat, respectively, WARS2-related parkinsonism and a DHPR deficiency.

## Time to Achieve a Molecular Genetic Diagnosis

**Figure 2:**
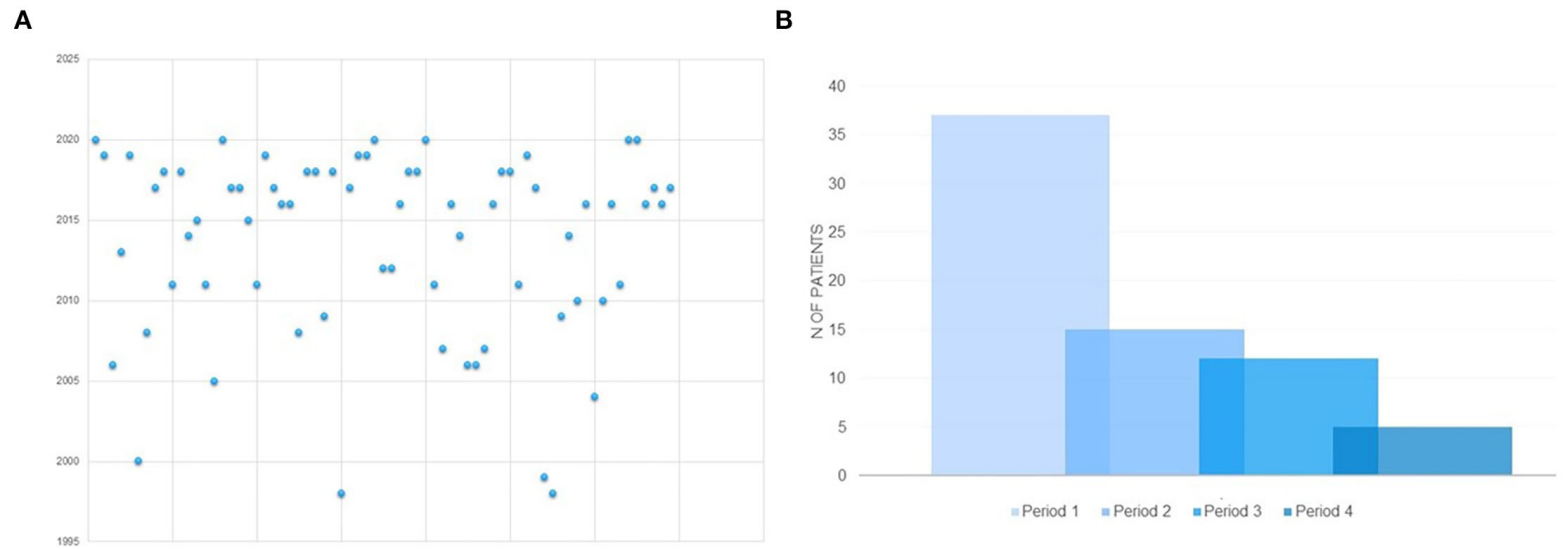
**(A)** Distribution of molecular genetic diagnosis per year in the whole sample. **(B)** Distribution of molecular genetic diagnosis according to temporal periods in which the molecular genetic diagnosis was made. PERIOD 1: 2016-2020; PERIOD 2: 2011- 2015; PERIOD 3 = 2006-2010; PERIOD 4 = before 2005.

The present study failed to demonstrate that specific combinations of seizure or movement disorder types might have been associated with an earlier etiological diagnosis (Tables 3, 5, 6). Diagnostic delay was longer in Group B than in Group A (Tables 3, 5). The analysis of the distribution of genetic diagnosis per year and diagnostic delay showed a higher gain in diagnostic yield of molecular genetic investigations in the last 5 years (Figures 2A,B).

**Table 5:** Differences between Group A and Group B in terms of diagnostic delay and temporal periods in which the molecular genetic diagnosis was made.

	**Period 1**		**Period 2**		**Period 3**		**Period 4**		**F**	***p-*value**
	**M**	**sd**	**M**	**sd**	**M**	**sd**	**M**	**sd**		
**Group A (*****N*** **=** **53)**										
Diagnostic delay (years)	6.86	7.52	5.26	6.22	3.18	2.28	3.60	1.73	0.933	0.432
*p <0.01
**Group B (*****N*** **=** **16)**										
Diagnostic delay (years)	23.15	13.32	3.1	4.13	4.67	4.07	-	-	8,126	0.005

*Period 1, 2016-2020; Period 2, 2011- 2015; Period 3, 2006-2010; Period 4, before 2005*.

**Table 6:** Differences in terms of age at diagnosis, age at the onset of movement disorders, and diagnostic delay according to the different hyperkinetic movement disorders that were observed in patients of Group A.

	**Ataxia**		**Dystonia**		**Stereotypies**		**Chorea**		**F**	***p*-value**
	**M**	**sd**	**M**	**sd**	**M**	**sd**	**M**	**sd**		
**Group A (*****N*** **=** **53)**										
Age at the diagnosis (years)	8.04	4.68	6.69	48,96	4.08	3.04	16.33	12,63	6,792	,001
Age at the onset of the movement disorders (years)	5.05	4.40	3.10	3.74	1.81	1.24	6.03	4.83	3,191	,032
Diagnostic delay (years)	5.26	3,18	5.20	5.14	3.05	3.38	15.27	12.12	7,573	,001

*Significance = p < 0.05*.

Analysis of variance and *post-hoc* tests with Bonferroni correction for multiple comparisons evidenced, in Group A, a significantly longer diagnostic delay and higher age at molecular genetic diagnosis in patients who presented with chorea at the onset if compared with the ones presenting with ataxia, dystonia, and stereotypies (Table 6).

## Discussion

The literature provides few epidemiological data about the distribution of molecular genetic diagnosis of developmental encephalopathies with epilepsy and movement disorders in the pediatric populations while several recent studies reported detailed and updated genotype-phenotype correlations for about 60 single-gene related disorders other than historically well-known patterns such as the ones of Rett or Angelman syndrome (1, 13–18). The studies including epidemiological data were often hardly comparable because of several methodological differences in terms of analyzed cohorts, inclusion and exclusion criteria, and modalities of data collection (1, 2). Most of these data were not statistically significant because of the rare or ultrarare prevalence of the analyzed diseases.

A recent systematic review of 49 papers identified 27 neonatal-onset single gene-related diseases presenting with severe epileptic and developmental encephalopathies and a predominance of hyperkinetic movement disorders in more than 85% of the cases, with neurometabolic conditions not included in the analysis (2). The proportion of neurometabolic diseases in a Canadian cohort of 197 patients referring to a single pediatric epilepsy center, who underwent targeted gene panels or whole-exome sequencing, accounted for 13% of cases (19). In the same cohort, a co-occurrent movement disorder was reported in almost one-fourth of the cases with a large predominance of dystonia (8,6% of the patients) (19). In a smaller Japanese cohort of 11 patients, 9 different monogenic diseases presenting with early-onset hyperkinetic movement disorders were diagnosed in 7 infants who had a West syndrome and in 2 children with a non-syndromic epileptic encephalopathy (20). Trump et al. (21) identified movement disorders in 4 out of 71 patients with a confirmed genetically determined epilepsy while Cordeiro et al. (22) reported an epileptic syndrome in 13 out of 21 patients with a genetically determined movement disorder.

The herein-reported single center retrospective cohort analysis depicted the heterogeneous clinical panorama and the diagnostic yield that may be observed in a specialistic setting.

The analysis of etiologies suggested a predominant association of chorea with a subset of genes or CNV involved in post-synaptic signaling (e.g., GNAO1, GNB1, FOXG1, MEF2C, CLN2, MeCP2 duplication) and a high quote of the pathogenic gene or chromosomal variants associated with neurodegenerative and metabolic diseases among patients presenting with myoclonus and parkinsonism.

Patients with hyperkinetic movement disorders, that were diagnosed in our center, presented with an earlier onset of epileptic seizures, a predominant generalized motor semiology of seizures at the onset, and prominence of multifocal EEG abnormalities. The prominent phenotypic features of patients with hypokinetic movement disorders included a later onset of seizures, a higher frequency of focal seizures at the onset, and focal EEG abnormalities.

The onset of seizures was noticed earlier than the onset of movement disorders in patients presenting with hyperkinetic movement disorders while the opposite occurred in patients with hypokinetic manifestations.

A possible explanation might be represented by an underdiagnosis of movement disorders because of their benign nature (e.g., stereotypes) or lack of awareness, vs. the higher social and clinical alarm induced by seizures. A second explanation could rely upon the different etiological spectrum observed in hyperkinetic and hypokinetic groups. In most patients from Group B (60 vs. 20% in Group A), epilepsy is the result or the by-product of a neurodegenerative process, and therefore it can appear later in the disease course, while in most patients from Group A (42/53) epilepsy is one of the first signs of a developmental and epileptic encephalopathy related to disfunction of genes involved in neurotransmission, neuronal excitability, or neural development.

A significant diagnostic delay was experienced in patients in which chorea represented the main movement disorder at the onset. This delay may result from different factors: (a) the highest tendency of physicians, especially if specific expertise in rare diseases lacks, to consider chorea mainly as a symptom of acquired diseases (e.g., Sydenham Chorea or other autoimmune disorders) with a possible underestimation of genetic etiologies; (b) the late diagnosis in patients carrying variants in novel disease-causing genes that were discovered up to several years after the onset of symptoms and the first clinical evaluation of patients in which pathogenic single gene variants or chromosomal aberrations were reported for the first time in the literature) (23).

The significantly longer diagnostic delay in Group B is probably due to the extremely rare occurrence of hypokinetic movement disorders in pediatric ages, especially in patients studied before the era of next generation sequencing. An extensive educational campaign focused on the peculiar phenotypic features and the availability of reliable biochemical (e.g., CSF neurotransmitter measurement in the disorders of monoamine metabolism or urinary copper in Menkes disease) and neuroimaging (e.g., cerebellar atrophy in Neuronal Ceroid Lipofuscinosis or lactate peak at HMRS in Menkes disease) biomarkers might increase the knowledge of genetically determined hypokinetic movement disorders among pediatric neurologists (13).

The limits of the study are strictly correlated with its retrospective design, the rarity of the explored diseases, and the heterogeneous spectrum of genetic etiologies while a bias might be represented by the inclusion of patients with an established molecular genetic diagnosis only (and the subsequent exclusion of patients with comparable spectra but without molecular genetic confirm). Moreover, this study did not systematically explore the longitudinal changes of epilepsy and movement disorder phenotypes from infancy into adulthood with a subsequent possible loss of useful information about prognostic implications even if these details are already available in the literature for some single-gene diseases (24–26). A further limit may be correlated with the current unavailability of an acceptable classification for movement disorders taking into account specific pediatric peculiarities (i.e., the high quote of mixed movement disorders, disorders of tone and postures, weakness, ataxia, and apraxia).

## Conclusions

This paper explored the presenting patterns of genetically determined encephalopathies with epilepsy and movement disorders and highlighted some relevant clinical and diagnostic issues including (a) a more frequent etiological role of abnormalities of genes/chromosomal regions involved in neurotransmission, neuronal excitability, or neural development in patients with hyperkinetic movement disorders; (b) a higher frequency of neurodegenerative courses and a longer diagnostic delay in patients with hypokinetic movement disorders.

## Data Availability Statement

The raw data supporting the conclusions of this article will be made available by the authors, without undue reservation.

## Ethics Statement

Ethical review and approval was not required for the study on human participants in accordance with the local legislation and institutional requirements. Written informed consent to participate in this study was provided by the participants' legal guardian/next of kin.

## Author Contributions

MM and SG contributed to the conception and design of the study and wrote the first draft of the manuscript. SC, AR, and LC contributed to data collection and data analysis and wrote some sections of the manuscript. VL revised the manuscript. All authors contributed to the manuscript and read and approved the submitted version.

## Conflict of Interest

The authors declare that the research was conducted in the absence of any commercial or financial relationships that could be construed as a potential conflict of interest.

## Publisher's Note

All claims expressed in this article are solely those of the authors and do not necessarily represent those of their affiliated organizations, or those of the publisher, the editors and the reviewers. Any product that may be evaluated in this article, or claim that may be made by its manufacturer, is not guaranteed or endorsed by the publisher.
